# Epigenetic modulation of human neurobiological disorders: Lesch-Nyhan disease as a model disorder

**DOI:** 10.3934/Neuroscience.2025005

**Published:** 2025-04-15

**Authors:** Khue Vu Nguyen

**Affiliations:** 1 School of Medical Imaging, Jiangsu Vocational College of Medicine, Yancheng 224005 Jiangsu, China; 2 Center for Molecular Biophysics, CNRS Orleans, 45071 Orleans, France

**Keywords:** alternative splicing (AS), Alzheimer's disease (AD), β-amyloid precursor protein (APP) gene, antisense drugs, epigenetics, epistasis between HPRT1 and APP genes, glycosyl-phosphatidylinositol (GPI) anchor, hypoxanthine phosphoribosyltransferase 1 (HPRT1) gene, hypoxanthine-guanine phosphoribosyltransferase (HGprt) enzyme

## Abstract

Epigenetics is the study of how cells control gene activity without changing the DNA sequence. Epigenetic changes affect how genes are turned on and off or expressed, and thus help regulate how cells in different parts of the body use the same genetic code. Errors in the epigenetic process can not only lead to abnormal gene activity or inactivity, but can also influence alternative splicing (AS) and could cause human diseases. Understanding of how epigenetic defects can affect human health, especially for neurological disorders, could suggest targets for therapeutic interventions. For such a purpose, the Lesch-Nyhan disease (LND) has been selected as a valuable model to study the genetic-epigenetic interplay, especially to explore the epistasis between the housekeeping hypoxanthine phosphoribosyltransferase 1 (HPRT1) and β-amyloid precursor protein (APP) genes. This review is structured as follows: we begin with an overview about the monogenetic neurological disorders associated with epigenetic changes; next, the current knowledge on HPRT1 and APP genes is provided; then, the epistasis between HPRT1 and APP genes related to the neurobehavioral syndrome in LND is described; and finally, we present the construction of expression vectors to study intermolecular interactions between the hypoxanthine-guanine phosphoribosyltransferase (HGprt) enzyme and APP in LND. Information obtained from such expression vectors would be useful for future directions to design therapies through epigenetic interventions.

## 1. Introduction

Genetics is the study of how genes and traits are passed down from one generation to the next. The genetic information stored in DNA represents the genotype, whereas the phenotype results from the “interpretation” of that information. Epigenetics describes factors beyond the genetic code. The word epigenetics means things imposed “on top of genetics” [1]. The epigenetic process includes DNA methylation, histone modification, and RNA-associated silencing. Errors in the epigenetic process can not only lead to abnormal gene activity or inactivity, but can also influence alternative splicing (AS) and could cause human diseases [2],[3].

In order to understand how epigenetic defects that affect human health could suggest targets for therapeutic interventions, especially for neurological disorders, the Lesch-Nyhan disease (LND) has been selected as a valuable model to study the genetic-epigenetic interplay, especially to explore the epistasis between the housekeeping hypoxanthine phosphoribosyltransferase 1 (HPRT1) and β-amyloid precursor protein (APP) genes. This review is structured as follows: we begin with an overview about the monogenetic neurological disorders associated with epigenetic changes; next, the current knowledge on HPRT1 and APP genes is provided; then, the epistasis between HPRT1 and APP genes related to the neurobehavioral syndrome in LND is described; and finally, we present the construction of expression vectors to study intermolecular interactions between the hypoxanthine-guanine phosphoribosyltransferase (HGprt) enzyme and APP in LND. Information obtained from such expression vectors would be useful for future directions to design therapies through epigenetic interventions.

## 2. Monogenetic neurological disorders affected by epigenetic changes and treatment overview

There are more than a dozen monogenetic neurological syndromes that are affected by changes in DNA methylation and histone modifications; for details, please refer to references [4]–[6].

### 2.1. Neurological disorders affecting DNA methylation

As an example, mutations and structural variants in the methyl CpG binding protein 2 (MECP2) gene that encodes the protein MECP2 are the major causes of Rett syndrome, a rare neurodevelopmental disorder (incidence 1 in 10,000). The MECP2 gene is X-linked and subject to X-inactivation. Rett syndrome occurs almost exclusively in girls, and is usually inherited in a dominant manner; boys who have a similar mutation typically die shortly after birth. The diagnosis is based on symptoms and can be confirmed with genetic testing. Those affected often have slower growth, difficulty walking, and a smaller head size (microcephaly; people with this medical condition often have an intellectual disability, poor motor function, poor speech, abnormal facial features, seizures, and dwarfism). The complications of Rett syndrome can include seizures, scoliosis (a medical condition in which a person's spine has an irregular curve in the coronal plan), and sleeping problems [7]. The MECP2 protein is found in all cells in the body, including the brain, and acts as a transcriptional repressor and activator, depending on the context. However, the idea that MECP2 functions as an activator is relatively new and remains controversial. The MECP2 protein is an important reader of DNA methylation. It is likely to be involved in turning off (repressing or silencing) several other genes. This prevents the genes from making proteins when they are not needed. Researchers have not yet determined which genes are targeted via the MECP2 protein; however, such genes are probably important for the normal function of the central nervous system (CNS).

### 2.2. Neurological disorders with histone defects

An example concerning histone defects is found in the case of Kleefstra syndrome, which is a rare genetic condition (incidence of 1: 25,000 to 1: 35,000) that affects multiple organ systems and has specific developmental and behavioral symptoms. Kleefstra syndrome is caused by either a deletion at 9q34.3 or mutations in the euchromatic histone-lysine N-methyltransferase 1 (EHMT1) gene, which is located in the same genomic region. EHMT1 encodes the protein EHMT1 (G9a-like protein, GLP). Kleefstra syndrome is inherited in an autosomal dominant manner [8]. Children with Kleefstra syndrome may have specific facial features including a small head size (microcephaly), a broad forehead, widely spaced eyes (hypertelorism), distinctive eyebrows, and large tongues (macroglossia). Additionally, heart, kidney, genital, and brain abnormalities may be seen. Most people with Kleefstra syndrome will have some form of intellectual disability, which may occur with autistic-like features. Diagnostic tests are either conducted at birth or later in early childhood [9],[10].

### 2.3. Epigenetic targets in the treatment of neurological disorders

Aberrant epigenetics resulting from DNA methylation (hypomethylation and hypermethylation) and histone modifications are potentially reversible. This possibility represents a promising field of epigenetic therapy. The main purpose of epigenetic drugs [1] resides in the inhibition of DNA methyltransferases (DNMTs, catalyze DNA methylation) such as analogues 5-azacytidine and 5-aza-CR, histone acetyltransferases (HATs, add acetyl groups to histones) such as C646, histone deacetylases (HDACs, remove acetyl groups) such as vorinostat, and histone methyltransferases (HMTs, catalyze histone methylation) such as tazemetostat. However, there are still some challenges related to the cell permeability, low bioavailability, selectivity, and toxicity, which limit the improvement from these epigenetic drugs and thus require additional investigation [11].

### 2.4. Conclusion

Most of the clinical trials with epigenetic drugs are applied in the fields of oncology and general medicine, and the treatment options for neurological disorders will soon be enriched. The findings from molecular mechanisms that explain the molecular physiology and medicine in rare monogenic neurological disorders could provide useful principles for other common and complex neurological disorders such as Alzheimer's disease.

## 3. HPRT1 gene and the Lesch-Nyhan disease - APP gene and Alzheimer's disease

### 3.1. HPRT1 gene and the Lesch-Nyhan disease overview

**Figure 1.**
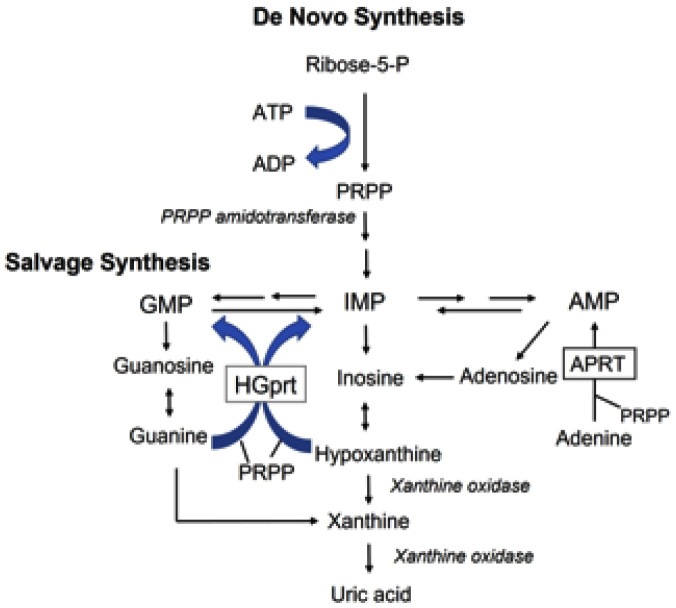
Purine metabolism. HGprt is the “salvage enzyme” for purines. This enzyme catalyzes the reversible transfer of the 5-phosphoribosyl group between α-D-5-phosphoribosyl-1-pyrophosphate or simply phosphoribosyl-pyrophosphate (PRPP) and a purine base (hypoxanthine, a modified form of adenine, or guanine) to either form a purine nucleotide inosine monophosphate (IMP) or guanosine monophosphate (GMP), respectively. Failure of this enzyme has two results: (1) cell breakdown products cannot be reused are degraded, which gives rise to an increased amount of uric acid, a purine breakdown product; and (2) the *de novo* pathway is stimulated due to an excess of PRPP.

There are many good reviews concerning the housekeeping hypoxanthine phosphoribosyltransferase 1 (HPRT1) gene (MIM: 308000) that encodes the hypoxanthine-guanine phosphoribosyltrnasferase (HGprt) enzyme and the resulting Lesch-Nyhan disease (LND, a rare X-linked recessive neurogenetic disorder) (MIM: 300322); please refer to references [12],[13]. Briefly, the HPRT1 gene is located on the long arm of the X chromosome (Xq26.2–q26.3). This gene has only one functional messenger RNA transcript (HPRT-mRNA) and contains 9 exons and 8 introns. HGprt is the “salvage enzyme” that works by utilizing previously made compounds in order to synthesize complex compounds for the generation of purine nucleotides though the purine “salvage pathway” (Figure 1). Mutations in the HPRT1 gene can lead to either a complete or severe deficiency of the HGprt activity, which results in an accumulation of guanine and hypoxanthine that oxidize in uric acid leading to hyperuricemia and cause the LND or Lesch-Nyhan variants (LNVs, MIM: 300323), respectively. Patients with LND are unable to walk and are confined to a wheelchair with hyperuricemia, gout, intellectual impairment, and self-mutilating behaviors. LNVs patients are characterized by an overproduction of uric acid and a variable spectrum of neurological manifestations without self-mutilating behaviors. Since the HPRT1 gene is on the X chromosome, males are affected and females in at-risk families may be carriers of the mutation. The prevalence of LND is about 1 in 380,000 live male births. This disease has been reported in most races, with a similar frequency in all populations.

The diagnosis of LND is based on the three following criteria: (1) a biochemical diagnosis by measuring the HGprt enzyme activity to determine the complete or severe deficiency of the HGprt enzyme activity; (2) a molecular diagnosis by detecting the presence of a mutation in the HPRT1 gene; and (3) a clinical examination by observing whether clinical symptoms are present or not.

Despite having been characterized 60 years ago (from the first report of Lesch M and Nyhan WL in 1964), there is no satisfactory explanation of how the loss of the HGprt enzyme function affects the brain to cause the intellectual impairment and self-mutilating behaviors in LND [12]. This has made the development of an effective treatment for LND difficult. Until now, there has been no effective drug that controls the associated self-mutilating behaviors. Allopurinol can be taken to control the amount of uric acid produced. S-Adenosylmethionine and Clonazepam can also be administered, though the symptoms of self-mutilating behaviors have been shown to not improve. In summary, the self-mutilating behaviors must be managed with a combination of physical restraints, behavioral, and pharmaceutical treatments such as elbow restraints, dental guards, and benzodiazepines. In order to develop more effective treatments for LND, the underlying etiology of the disease must be understood.

### 3.2. APP gene and Alzheimer's disease overview

**Figure 2.**
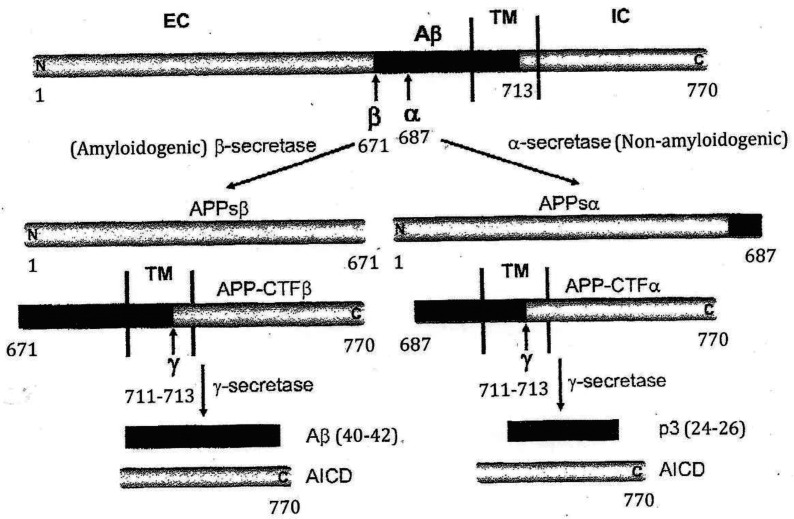
Schematic diagram of APP processing pathways (not drawn to scale). The β-amyloid (Aβ) domain is highlighted in black. For simplicity, only one cleavage site is shown for each enzyme, except for γ-Secretase with γ_40_ and γ_42_ cleavage sites. EC: extracellular domain; TM: transmembrane domain; IC: intracellular domain. The amino-acid sequence of the Aβ region is shown along with the secretase cleavages sites: BACE1 cleaves APP after Met_671_ (β), whereas a disintegrin and metalloprotease-10 (ADAM10, best candidate α-secretase) processes APP within the Aβ peptide sequence after Lys_687_ (α), thus generating the p3 peptide (non-amyloidogenic). γ-secretase cleavage in the transmembrane region (TM) involves presenilin 1 (PS1) and generates Aβ peptides of mainly 40 and 42 amino acid residues, namely long (γ_40_) and (γ_42_), respectively (amyloidogenic).

Regarding APP, this protein is ubiquitously expressed in a broad spectrum of cell types including neuronal and non-neuronal cells [14], while the nature of APP has been mainly studied in neuronal cells due to its pathological significance in Alzheimer's disease (AD); for details, please refer to references [15]–[18]. Briefly, the housekeeping APP gene (MIM: 104760) that encodes the APP is located on chromosome 21 (21q21.2–3), spans approximately 240 kb, and contains 18 exons. It is a type I transmembrane protein composed of a long N-terminal extracellular domain (EC), an intracellular domain (IC) with a short C-terminal cytoplasmic region, and a short transmembrane domain (TM) (Figure 2). Under physiological conditions, the full-length APP is processed by at least three proteinases, termed α, β, and γ-secretases (Figure 2). Until now, the physiological function of the entire APP and its cleavage products remains largely unclear.

AD is a degenerative brain disorder and is the most common form of dementia. The pathology in AD is characterized by the extracellular deposition of amyloid plaques of amyloid-β (Aβ) peptides (senile plaques, SPs) that originated from the proteolysis of APP between neurons in the brain and intracellular aggregates of neurofibrillary tangles (NFTs) of hyperphosphorylated tau proteins inside the neurons. AD is manifested by a decline in faculties such as memory, language, problem-solving, and other cognitive skills that affect a person's ability to perform everyday tasks. In AD (and other forms of dementia), the hippocampus is one of the first regions of the brain to suffer damage, in which short-term memory loss and disorientation are among the early symptoms. There are two types of AD: sporadic AD (SAD, onset age >65 years) and familial AD (FAD, onset age<65 years). SAD accounts for ~99% of AD cases, while FAD accounts for ~1% of cases. Mutations in the APP gene and the presenilin (PS) genes PS1 and PS2 are known to be associated with FAD. AD includes memory loss and the potential for death. Dementia is currently the sixth-leading cause of death among all diseases. Owing to the dramatic increase in the population as the year and age progresses, AD is often engaged as life threatening and an economic and health care burden.

Currently, the diagnostic criteria from the National Institute of Neurological and Communicative Disorders and Stroke and the Alzheimer's disease and Related Disorder Association (NINCDS-ADRDA) are used to identify patients with AD who have overt dementia corresponding to a neuropathologically advanced disease. Consequently, early recognition of the disease needs to be improved. However, currently, none of the imaging, genetic, or biochemical markers have been sufficiently qualified as a surrogate for the actual NINCDS-ADRDA.

Although the amyloid cascade hypothesis has been offered as a broad framework to explain the AD pathogenesis, certain observations do not easily fit with the simplest version of the hypothesis [15]–[18]. For now, clinically validated treatments for AD remain confined to symptomatic interventions such as treatments with acetylcholinesterase inhibitors such as donepezil, the β-site APP cleaving enzyme 1, BACE1, β-, γ-secretases inhibitors, active and passive immunotherapies to inhibit the Aβ production or decreasing the brain Aβ load, and drugs that ameliorate behavioral disturbances such as antidepressants, sleeping tablets, and tranquilizers. However, no treatments that slow the cognitive decline has been documented in humans to date [15]–[18].

## 4. Epistasis between HPRT1 and APP genes related to the neurobehavioral syndrome in LND/LNVs

Currently, histopathological studies of autopsy tissues from LND brains patients have revealed no signs suggestive of a degenerative process or other consistent abnormalities in any brain region [19]. However, the following points have been documented:

-Reduced brain volumes: reductions of white (26% in LND and 14% in LNV) and gray (17% in LND and 15% in LNV) have been seen [20]–[23]; therefore, all patients with LND and many with LNV have generalized dystonia, thus limiting their ability to walk;

-AD undergoes gene expression aberrations in the purinergic dysregulation of HGprt deficiencies [24];

-Expression of the APP gene from human skin fibroblasts from normal subject (control) and LND/LNVs patients occurred as follows: (a) under epigenetic regulation of alternative APP pre-mRNA splicing; and (b) via epistasis between HGPRT1 and APP genes that affect alternative APP pre-mRNA splicing, thus leading to the production of alternative APP fragments that might be responsible for the difference in severity for LND/LNVs patients [25],[26];

-APP is an important developmental gene for brain morphology and function [27]. Additionally, APP and APP-like protein-2 (APLP2) have been suggested to act as tumor growth factors in the pathogenesis of several somatic tissue cancers, and they may be used as potential biomarkers to guide clinical diagnoses and the treatment of cancers [28]–[30]. Interestingly, the overexpression of both histone deacetylase and APP in hepatocellular carcinoma have been reported [29];

-Development of thrombosis from some LND/LNVs patients in which hypercoagulability from red blood cells has been reported [31]–[34], while APP is an important regulator of vein thrombosis and controls coagulation [35]. These findings suggest that the APP pathway could be implicated in the development of neurological features and thrombotic events of LND/LNVs;

-Elevated expression of HPRT1 in malignant tissues of several types of cancers [36]–[41]. Additionally, the surface expression on the plasma membrane of the HGprt enzyme in several somatic tissue cancers was reported [42]–[48]. Therefore, HGprt can be used as a surface antigen for targeted immunotherapies, and HPRT1 can be used as a biomarker for the detection and treatment of cancer. All these findings suggest a potential molecular link between APP and HGprt in LND and cancer [49].

Currently, more than 600 mutations from the HPRT1 gene responsible for this disease have been reported [12],[13]. At present, one of the controversial aspects of the disease is the relationship between the genotype and phenotype such as cases of patients lacking a mutation in the coding region of the HPRT1 gene and families who despite sharing the same genetic defect show disorders with differing severity [50]–[53]. One possible explanation is that there is a second gene that may cause clinical features that closely resemble LND. However, this possibility is unlikely because the clinical phenotype of LND has never been associated with any other gene [53]. Another possibility is that epigenetic processes, which modify the genetic expression without changing the sequence of the DNA, could explain the clinical variability observed in this disease [54],[55]. So far, there are no proven examples of epigenetic abnormalities that involve DNA methylation, histone modification, and micro RNAs-associated silencing for LND [55]. Even so, this epigenetic modality alone [54],[55] would be not satisfactory to explain the whole aspects of the disease such as hypercoagulability with the presence of elevated levels of proteins involved in coagulation and hemostasis in red blood cells, thus eventually leading to thrombotic events [31]–[34]. Otherwise, it is important to note that the human genome is estimated to contain 20,000 to 25,000 genes that encode proteins essential for life [56]; a gene does not function in isolation, but rather acts with other genes in a network to influence complex traits of complex disorders. There are probably other undiscovered factors other than the genotype at the HPRT1 locus, such as modifier genes and environmental factors that act in the phenotypic expression of the disease associated with HGprt deficiency. Then, this issue concerns the epigenetic modifications in epistasis (gene-gene interactions) and/or gene-environment interactions. Here, APP could be a modifier gene in which its expression has been found, for the first time, under epigenetic regulation in LND (via epistasis affecting alternative APP pre-mRNA splicing and producing alternative APP fragments that might be responsible for the syndromes in LND/LNVs [25],[26]). To identify the most abundant alternative APP fragments that might be responsible for the syndromes in LND/LNVs, a report on the quantification of various APP-mRNA isoforms in biological samples has been described [57].

The cross-talk among genes in a network plays an important role in signaling pathways associated to the regulation of intracellular protein transport. This cross-talk among genes could influence complex traits of disorders such as AD and cancer. One example of cross-talk between proteins involves the cross-talk between mutant p53 and p62/SQSTM1 that augments cancer cell migration by promoting the degradation of cell adhesion proteins [58]. Currently, there is no information from the literature concerning the molecular cross-talk between HPRT1 and APP genes. It is evident that APP is not special in this regard as a modifier gene, and many unknown genes could have their AS altered, both qualitatively and quantitatively, due to HGprt deficiency, though this is currently untested. Moreover, there have been no studies of brain transcriptomes or metabolomes from LND patients to confirm the scope of homeostatic perturbations. Even so, it is very difficult to make a conclusion from such studies when we do not have any idea about the potential gene candidate of the epistatic effects. Furthermore, there are different difficulties in detecting and characterizing epistasis, such as challenges of modeling non-linear interactions, and in the interpretation of results [59]–[63]. Elucidating how APP interactions contribute to normal neuronal functions offers insights into how APP may directly contribute to the pathogenesis of several neurological diseases [64]. In a way, these findings [25],[26] were the first demonstration of how such epigenetic defects (potentially reversible) could provide a target for therapeutic interventions through the AS process of APP gene observed from LND, while the impacts of APP on different human diseases such as neurodevelopmental and neurodegenerative disorders, metabolic disorders, diabetes, obesity, and cancer have been reported in the literature. For clarification of the epistatic effects related to the APP and HPRT1 genes in LND/LNVs, the construction of expression vectors for the HGprt enzyme and APP is performed to study the intermolecular interactions.

## 5. Construction of expression vectors for HGprt enzyme and APP

**Figure 3.**
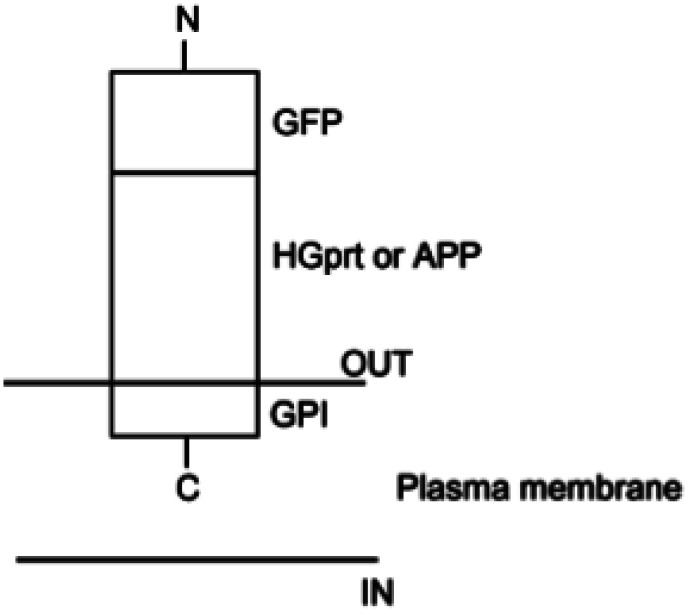
Schematic representation of the membrane topology of the expression vectors for HGprt and APP. The mammalian expression vector pcDNA™ 3.1 (+) is used as the backbone in which all genes of interest are inserted in the right frame into the pcDNA™ 3.1 (+) vector. The construct is comprised of the sequence encoding the C-terminal of the glycosyl-phosphatidylinositol, GPI, anchor derived from the human folate receptor 1 (FOLR1) protein and the entire coding sequence (CDS) of either hypoxanthine phosphoribosyltransferase 1 (HPRT1) gene, which encodes the HGprt enzyme, or the CDS of APP gene, which encode APP coupled with the CDS of the green fluorescent protein (GFP) gene.

For details on the construction of expression vectors for the HGprt enzyme and APP (Figure 3), please refer to reference [65].

These expression vectors can be used as tools for the following:

(a) studying the effects of mutations on the HGprt enzyme found from different LND/LNVs patients (i.e., exploring intermolecular interactions between the mutated HGprt enzyme and its substrates: α-D-5-phosphoribosyl-1-pyrophosphate (PRPP) and a purine base, either guanine or hypoxanthine, a modified form of adenine);

(b) studying the effects of the surface expression of the HPRT1 gene in cancer (i.e., exploring intermolecular interactions between HGprt and other molecules of interest, as well as the ones for targeted immunotherapies);

(c) studying intermolecular interactions between HGprt and other molecules of interest, which would also allow to one to explore the mechanism that links HGprt deficiencies, purinergic pathways, and neural dysfunctions of LND;

(d) exploring the structure and physiologic function of APP (or APLP2) by investigating intermolecular interactions between APP and its cleavage products and other molecules of interest. This study is important because all fragments of APP, including the Aβ peptide, are part of normal the physiology [28],[30],[64],[66]–[69]; targeting the components of APP processing as a pharmacologic strategy will not be without consequences;

(e) studying intermolecular interactions between APP and the HGprt enzyme to explore the epistasis between the housekeeping HPRT1 and APP genes. Findings from such a study will provide useful principles for other common and complex disorders such as AD and cancer;

(f) application in human skin fibroblasts and antisense therapies. Human skin fibroblasts could be used as tools for the elucidation of pathomechanisms, thus leading to the manifestation of neurological diseases including those produced by LND, AD, etc.; consequently, they can offer strategies for disease modeling and drug discovery such as antisense therapies in central nervous system disorders [70]–[73];

Additionally, these expression vectors can be used as a model for the construction of expression vectors for any protein that targets the cell plasma membrane via GPI anchor; therefore, they could be useful in the following:

(a) contributing to novel approaches for the treatment of neurodegenerative diseases and cancer related to the prion protein. Indeed, the cellular prion protein (PrP^c^) is mostly located at the cell surface (attached to the plasma membrane via a GPI anchor), and is highly expressed in both the nervous and immune systems as well as in other organs [74],[75]. However, its function in the transmembrane signal transduction has been unclear [74],[75]. Misfolding of PrP^c^ is associated with the transmissible spongiform encephalopathies (TSEs), while its normal PrP^c^ conformer serves as a receptor for oligomers of the Aβ, and its overexpression has been found in different cancer cell lines [74],[75];

(b) investigating the molecular mechanisms behind the mechanotransduction process from the muscle LIM protein (MLP), which is believed to be involved in human heart failure [76];

(c) studying the regulation of the immune system via the human leukocyte antigen (HLA) complex in which HLA genes are highly polymorphic with numerous alleles, thus allowing the body to regulate the adaptive immune system and to differentiate self-cells and non-self-cells through cell-surface proteins [77];

(d) selecting anti-cancer medication to use in immunotherapies via the immune checkpoint inhibitor (ICI) therapy, thus allowing cytotoxic T cells of the adaptive immune system to be capable of killing cancer cells [78]–[80];

(e) selecting antiviral drugs and developing vaccines for the severe acute respiratory syndrome coronavirus 2 (SARS-CoV-2), also known as coronavirus disease 2019 (COVID-19 virus), that caused the most recent global pandemic [81]–[83];

(f) studying gene-environment interactions to investigate the potential impact of any chemical compounds on any genes of interest [84]. The development of carbonic anhydrase (CA)-based CO_2_-capture would be a very interesting tool to combat the current global warming crisis related to climate change (environmental application) [85]–[87]. The role of CA in cell proliferation and oncogenesis can also be studied, as well as designing CA's inhibitors or activators (biomedical applications) [88].

## 6. Conclusion

Epistasis is a phenomenon in genetics in which the effect of a gene mutation is dependent on the presence or absence of mutations in one or more other genes, namely modifier genes. In other words, the effect of the mutation is dependent on the genetic background in which it appears. Therefore, epistatic mutations have different effects on their own as compared to when they occur together. Thus, the effects of a given gene on a biological trait are either masked or enhanced by one or more other genes. The phenomenon arises due to interactions, either between genes (such as mutations also being needed in regulators of gene expression) or within them (multiple mutations being needed before the gene loses function), thus leading to non-linear effects. Epistasis has a great influence on the shape of evolutionary landscapes, which leads to profound consequences for evolution and the evolvability of phenotypic traits. The present manuscript aimed to enhance the importance of an epigenetic modulation via epistasis between APP and HGprt in LND for the first time based on findings found from various publications, thus suggesting that the pathogenesis of this monogenic LND results from combinatorial and multigenic defects and could be considered as a model disorder for the research of other genetic diseases, especially human neurological disorders.

## Use of AI tools declaration

The authors declare they have not used Artificial Intelligence (AI) tools in the creation of this article.
